# Individual differences in EPA and DHA content of Atlantic salmon are associated with gene expression of key metabolic processes

**DOI:** 10.1038/s41598-019-40391-2

**Published:** 2019-03-07

**Authors:** Siri S. Horn, Anna K. Sonesson, Aleksei Krasnov, Hooman Moghadam, Borghild Hillestad, Theo H. E. Meuwissen, Bente Ruyter

**Affiliations:** 1 Nofima (Norwegian institute of Food, Fisheries and Aquaculture research), PO Box 210, N-1432 Ås, Norway; 2 Department of Animal and Aquaculture Sciences, Norwegian University of Life Sciences, N-1430 Ås, Norway; 3 SalmoBreed AS, Sandviksboder 3A, N-5035 Bergen, Norway

## Abstract

The aim of this study was to explore how individual differences in content of the omega-3 fatty acids EPA and DHA in skeletal muscle of slaughter-sized Atlantic salmon, are associated with expression of genes involved in key metabolic processes. All experimental fish were fed the same diet throughout life and fasted for 14 days prior to slaughter. Still, there were relatively large individual variations in EPA and DHA content of skeletal muscle. Higher DHA content was concurrent with increased expression of genes of the glycolytic pathway and the production of pyruvate and lactate, whereas EPA was associated with increased expression of pentose phosphate pathway and glycogen breakdown genes. Furthermore, EPA, but not DHA, was associated with expression of genes involved in insulin signaling. Expression of genes specific for skeletal muscle function were positively associated with both EPA and DHA. EPA and DHA were also associated with expression of genes related to eicosanoid and resolvin production. EPA was negatively associated with expression of genes involved in lipid catabolism. Thus, a possible reason why some individuals have a higher level of EPA in the skeletal muscle is that they deposit - rather than oxidize - EPA for energy.

## Introduction

Farmed Atlantic salmon has traditionally been very rich in the healthy long-chain omega-3 polyunsaturated fatty acids (LC n-3 PUFA) eicosapentaenoic- (EPA) and docosahexaenoic (DHA) acid, and thereby they are a valuable source of these fatty acids in the human diet. Limited availability of fishmeal and fish oil on the world market, in conjunction with a growing aquaculture industry, has led to a substantial substitution of marine ingredients with plant ingredients in feed for farmed salmon in the last few decades^1^. Consequently, decreased levels of EPA and DHA in farmed salmon tissues and organs are reported in Norwegian, Scottish and Tasmanian Atlantic salmon^1–3^. This is not only reducing the nutritional value of Atlantic salmon fillet to consumers, but may also influence fish health and quality^4^.

The LC n-3 PUFA content of fish tissues is a complex trait influenced by feed, life stage, and metabolic processes including digestibility and uptake of fatty acids (FA) in the gut, as well as selective transport, deposition and β-oxidation in organs and tissues^4–8^. In addition, endogenous omega-3 bioconversion enables salmonids to convert the shorter chain alpha-linolenic acid (ALA) to the longer chain PUFAs EPA and DHA through a series of desaturation and elongation steps, followed by peroxisomal β-oxidation^9,10^. In Atlantic salmon, the rate of bioconversion is inversely related to the amounts of fish oil in the diet^9,11–13^. The omega-3 bioconversion is also influenced by water temperature, the genetic background, and life stage of the fish^14,15^.

Both EPA and DHA fulfill numerous biological functions in the animal body. EPA and DHA are to some extent metabolically interchangeable making it in some cases difficult to pinpoint functions specific to each of them. However, it is known that DHA is more critical than EPA as a structural component of cell membranes including lipid rafts, while EPA is believed to play a more central role in regulation of several processes related to immunity and inflammation^16,17^. Atlantic salmon fed only EPA but no DHA developed abnormal intestinal morphology, while the fish fed only DHA but no EPA developed a normal intestine^4^, pointing to the importantce of DHA for functional intestinal cell membranes. Other studies have shown that EPA content of heart and head kidney tissues of Atlantic salmon is associated with reduced severity of the inflammatory response to the viral disease Heart and skeletal muscle inflammation^18^. Specific functions of EPA and DHA in production of eicosanoids and their role in inflammatory responses has been described both in fish^19^ and in mammals^20^. Further, different pro-resolving lipid mediators, resolvins and protectins, can also be generated. Resolvins are derived from both EPA and DHA, categorized as either E-series (from EPA) or D-series (from DHA), while protectins are derived from DHA^21,22^. Although little is known for fish, the different resolvins and protectins are shown to have potent specific immunoregulatory actions in mammals^21,22^.

Evidence from mammalian studies further indicates that tissue contents of EPA and DHA play important roles in the regulation of carbohydrate metabolism. Increased contents of EPA and DHA are reported to improve glucose uptake^23,24^, have favorable effects on glucose utilization^25^, and thereby have a preventive effect on development of metabolic syndrome^26,27^. EPA and DHA may also improve skeletal muscle capability of switching between use of lipids, protein and glucose for production of energy^28^. High levels of EPA may result in downregulated lipogenesis and upregulated mitochondrial β-oxidation^29^. The systems that regulate lipogenesis and LC n-3 PUFA biosynthesis in mammals are largely conserved in Atlantic salmon^30^, and dietary LC n-3 PUFA levels have been shown to influence lipid metabolism also in Atlantic salmon^31,32^. With the shift in salmon diets causing major alterations in EPA and DHA content of skeletal muscle, it is especially important to gain knowledge on how these alterations influence lipid and carbohydrate metabolic pathways.

Most gene expression studies of omega-3 effects in Atlantic salmon have focused on the liver transcriptome and its responses to changed dietary levels of LC n-3 PUFAs^13,33–36^. Skeletal muscle is highly relevant as it constitutes the largest part of the Atlantic salmon body, and expresses desaturase and elongase genes^37,38^, as well as transcription factors known to regulate lipid metabolism genes^8^. Further, there are inherent differences in the muscle content of EPA and DHA between Atlantic salmon fed the same diet^39^, but the metabolic differences between individuals with high and low levels remain unknown.

The main aim of this study is to investigate how individual differences in EPA and DHA content in skeletal muscle of Atlantic salmon are associated with expression of genes involved in key physiological processes, particularly nutrient metabolism. We limit this study to the contents of EPA and DHA in the skeletal muscle and transcriptome of skeletal muscle and liver.

## Methods

### Fish populations and recordings

The fish studied originated from families and documentation groups from the entire 2014 year-class of the Atlantic salmon breeding population of SalmoBreed AS. The fish were transferred to sea at a mean weight of 0.1 kg, and slaughtered approximately 12 months later, at a mean weight of 3.6 kg. The fish were fed a commercial broodstock feed from Skretting (https://www.skretting.com/en/products/atlantic-salmon/?lifephase=474980) with a high fish oil content, and were fasted 13–14 days prior to slaughter. All the fish were reared under the same conditions.

Skeletal muscle and liver tissue samples for RNA-sequencing were taken from each individual fish at harvest, immediately frozen in liquid nitrogen, and subsequently stored at −70 °C. Skeletal muscle samples for lipid and FA analysis were taken from Norwegian Quality Cut collected at harvest, frozen and stored at −20 °C. In total, 668 fish were analyzed for skeletal muscle FA composition. The ranges in bodyweight and fat content of skeletal muscle were 1.2–6.4 kg and 5.5–27.7%, respectively. In order to minimize the effects of size and lipid deposition, 59 fish were selected for RNA-sequencing based on bodyweight (3.3 to 3.9 kg) and skeletal muscle fat level (16 to 25%). The selected individuals came from 48 full sib families, originating from 39 sires and 48 dams.

### Lipid and fatty acid analysis

Total lipids were extracted from homogenized liver and skeletal muscle samples of individual fish, according to the Folch method^40^. Using one milliliter from the chloroform-methanol phase, FA composition of total lipids was analyzed following the method described by Mason and Waller^41^. The extract was dried briefly under nitrogen gas and residual lipid extract was trans-methylated overnight with 2′,2′-dimethoxypropane, methanolic-HCl, and benzene at room temperature. The methyl esters formed were separated in a gas chromatograph (Hewlett Packard 6890; HP, Wilmington, DE, USA) with a split injector, using an SGE BPX70 capillary column (length 60 m, internal diameter 0.25 mm, and film thickness 0.25 μm; SGE Analytical Science, Milton Keynes, UK) and a flame ionization detector. The results were analyzed using HP Chem Station software. The carrier gas was helium, and the injector and detector temperatures were both 270 °C. The oven temperature was raised from 50 to 170 °C at the rate of 4 °C/min, and then raised to 200 °C at a rate of 0.5 °C/min and finally to 240 °C at 10 °C/min. Individual FA methyl esters were identified by reference to well-characterized standards. The content of each FA was expressed as a percentage of the total amount of FAs in the analyzed sample. Presentation of the results will focus on the most abundant LC n-3 PUFAs of the fillet: 20:5n-3 (EPA) and 22:6n-3 (DHA), and the sum of EPA and DHA (EPA + DHA).

### Gene expression analysis

Total RNA was extracted from liver and skeletal muscle tissues of each individual fish using the PureLink Pro 96 RNA Purification Kit (Invitrogen), according to the manufacturer’s instruction. RNA was treated with PureLink On-Column DNase Digestion (Invitrogen) to remove any contaminating DNA. Samples were shipped to The Norwegian High-Throughput Sequencing Centre, where the mRNA library preparation and sequencing of transcripts were performed using standard protocols (www.illumina.com). Samples were sequenced on an Illumina HiSeq platform as paired-end 151 bp reads. After final quality control, results from 48 liver- and 59 skeletal muscle samples remained for analyses.

Processing of reads, alignment and annotation was performed according to Moghadam *et al*.^42^. Expression data were normalized via the median of the geometric means of fragment counts across all sample^43^. Cufflinks and Cuffdiff were used to estimate the expression abundances of the assembled genes and transcripts^44^.

### Statistical analysis

Gene expression data were normalized by calculating the aligned fragments per kilobase per million mapped fragments (FPKM). Normalized gene expression data were log2 transformed prior to the statistical analysis.

Trait-associated genes were defined by using linear regression analysis, testing for an association between continuous traits and mRNA expression. The FA content was considered the response variable and each individual gene expression an explanatory variable in the model. As suggested by Seo *et al.*^45^, univariate analyses were carried out for each FA trait. The following general linear mixed model was fitted for each trait:

$${{\rm{Y}}}_{{\rm{jk}}}={\rm{\mu }}+{{\rm{sex}}}_{{\rm{j}}}+{\rm{fat}}+{{\rm{gene}}}_{{\rm{k}}}+{\rm{family}}+{\rm{e}}$$

where *Y* is the skeletal muscle FA content (EPA, DHA or EPA + DHA) of one individual, *μ* the overall mean, *sex*_*j*_ the fixed effect of sex (male or female), *fat* the percentage of fat in the tissue of the gene expression as a covariate (muscle or liver), *gene*_*k*_ gene expression level of gene k, *family* random effect of family (1–48), and *e* random residual variation.

Genes were considered significantly associated with traits when the p-value of the regression coefficient was < 0.05. Genes of interest are presented with their regression coefficients. All significant genes are presented in Supplementary Table S1.

### Enrichment analysis

A search for enriched GO classes and KEGG pathways in the list of trait-associated genes was performed by counting of genes among the trait-associated genes and all genes that passed quality control. Enrichment was assessed with Yates’ corrected chi square test (p < 0.05). Terms with less than five genes were not taken into consideration.

The enrichment analysis was used for an initial screening to outline the functional groups and pathways of interest. Next, we focused on individual genes, taking into account the regression coefficients as metrics of expression differences between the phenotypes.

### Ethics statement

The study was based on *post mortem* sampling of material from fish harvested from a commercial breeding program for other purposes. The experimental plan was evaluated towards the Norwegian regulation for use of animals in experiments (FOR-2015-06-18-761, Forskrift om bruk av dyr i forsøk). The regulation states that activities related to non-experimental aquaculture activities are exempt from the regulation, and that it is legal to sample from animals *post mortem* without a specific license. Thus, the samples used in this study were collected in accordance with the Norwegian legislation for animal experiment.

## Results and Discussion

### Description of phenotypes of experimental fish

In this study, we investigated how individual differences in EPA and DHA content (% of total FAs) in skeletal muscle of Atlantic salmon were associated with muscular and hepatic expression of genes involved in key physiological processes, particularly nutrient metabolism. The fish originated from the same strain of a breeding population, were reared under the same conditions and were all fed the same diet (as described previously in Horn *et al.*^37^), and fasted for two weeks prior to slaughter.

The EPA and DHA content of skeletal muscle changes with fish size and fat content, therefore fish of approximately the same size (3.6 kg) were selected for the gene expression analyses, as shown in Table 1. The mean skeletal muscle fat content was 19.7%. The mean contents (in percentage of total FAs) of EPA and DHA were 5.8 and 6.6%, respectively (Table 1). This is approximately twice as high as the EPA and DHA content found in today’s commercially produced Atlantic salmon in Norway, which reflects the high levels of these FA in the feed used in the current study^4,46^. Standard diets for Atlantic salmon breeding populations are based on high levels of marine ingredients, usually a ratio of 70/30 between marine and plant ingredients, and the diets are therefore rich in EPA and DHA. Commercial diets, on the other hand, are based on 70% plant ingredients and are therefore low in EPA and DHA^1^.

**Table 1:** Descriptive statistics of the fish material.

Trait	N	Mean	SD	Min	Max
Bodyweight (g)	59	3634	161	3330	3890
Muscle fat (%)	59	19.7	1.99	16.2	25.3
EPA (%)*	59	5.81	1.46	4.36	8.79
DHA (%)*	59	6.63	0.49	5.75	7.45
EPA + DHA (%)*	59	12.45	1.60	10.35	15.71

N: Number of observations, SD: Standard deviation, Min: Minimum value. Max: Maximum value. *% of total FAs in skeletal muscle.

Relatively large variations in EPA and DHA contents were found in the skeletal muscle of the 59 experimental fish, even though the fish were of approximately the same size, reared under the same experimental conditions and fed the same diet throughout their life. The contents of EPA varied between 4.4 to 8.8% of total FAs, while DHA varied from 5.8 to 7.5% (Table 1). This indicates that the genetic variation between individuals may cause metabolic differences in one or several of the processes; dietary uptake, deposition, utilization or bioconversion of dietary fatty acids, and thereby result in individual differences in EPA and DHA contents of muscle despite the fact that the fish has been fed the same diet. Genetic variation was expected in the current study considering the family structure (48 full-sib families and 39 half-sib families). It has previously been shown that both EPA and DHA content of skeletal muscle are heritable traits^34,37^.

**Figure 1:**
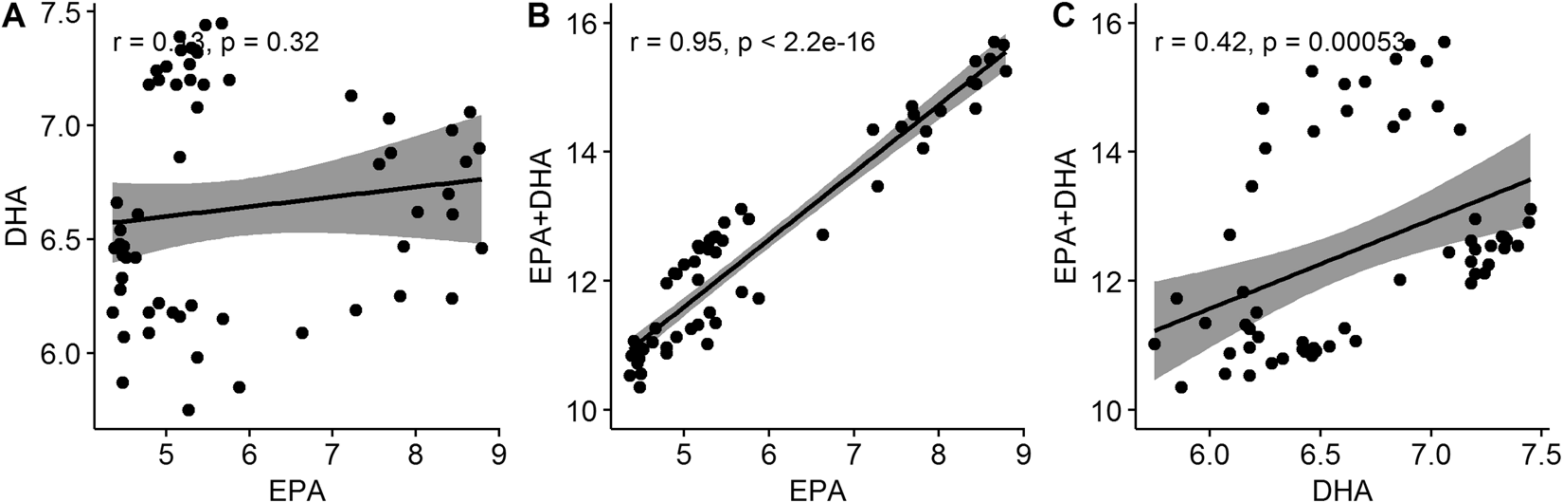
Correlation between contents of fatty acids in skeletal muscle. (**A**) Correlation between EPA and DHA (% of total FAs). (**B**) Correlation between EPA and EPA + DHA. (**C**) Correlation between DHA and EPA + DHA.

There was a low correlation between EPA and DHA percentage in skeletal muscle (Fig. 1A). A similar correlation was found in an analysis of a larger dataset of the same population (r = 0.23, n = 668)^37^. The fish with the highest DHA percentage had a low EPA percentage (Fig. 1A), indicating that some individuals had higher capacities for the conversion from EPA to DHA, or higher β-oxidation rate of one of the two FAs. EPA + DHA was more strongly correlated to EPA than DHA (Fig. 1B,C), because there was greater variation in EPA content (Table 1).

**Figure 2:**
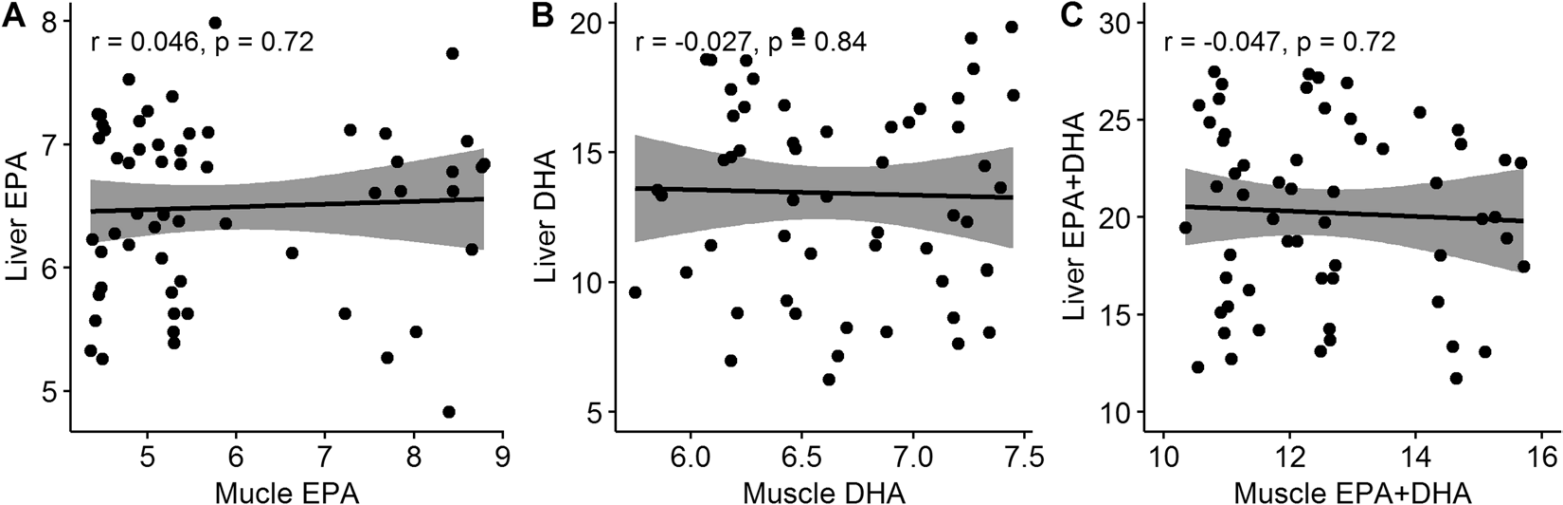
Correlation between liver and skeletal muscle content of fatty acids. Correlation between liver and skeletal muscle content of (**A**) EPA, (**B**) DHA, and (**C**) EPA + DHA (% of total FAs).

Correlations between liver and skeletal muscle EPA and DHA content were very low (Fig. 2). This is most likely due to the differences between liver and skeletal muscle lipid metabolism. Feed trials with Atlantic salmon have shown that the tissues respond differently to increased dietary levels of EPA and DHA and that the skeletal muscle FA composition is to a much higher extent than the liver influenced by dietary FA composition^4^.

### Gene expression differences between EPA and DHA

**Figure 3:**
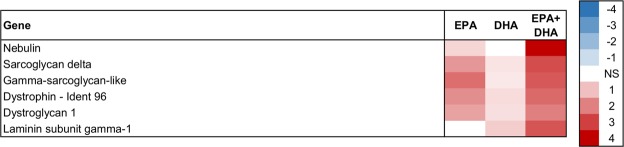
Skeletal muscle tissue development. Association between EPA, DHA or EPA + DHA content in muscle and expression of genes in skeletal muscle. Color scale shows linear regression coefficients. NS = no significant association.

**Figure 4:**
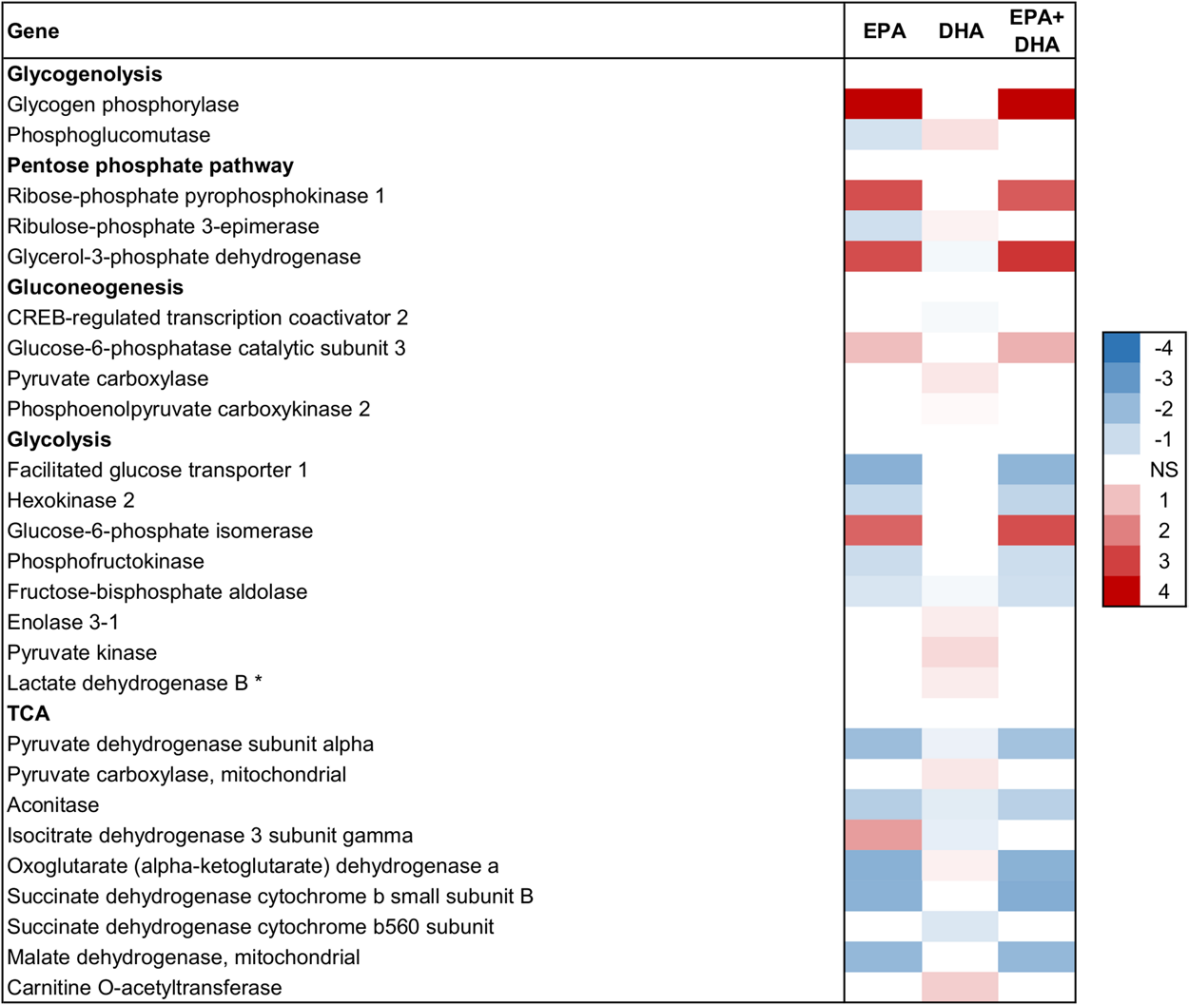
Carbohydrate metabolism in skeletal muscle. Association between EPA, DHA or EPA + DHA content in muscle and expression of genes in skeletal muscle. Color scale shows linear regression coefficients. NS = no significant association. *Anaerob glycolysis.

**Figure 5:**
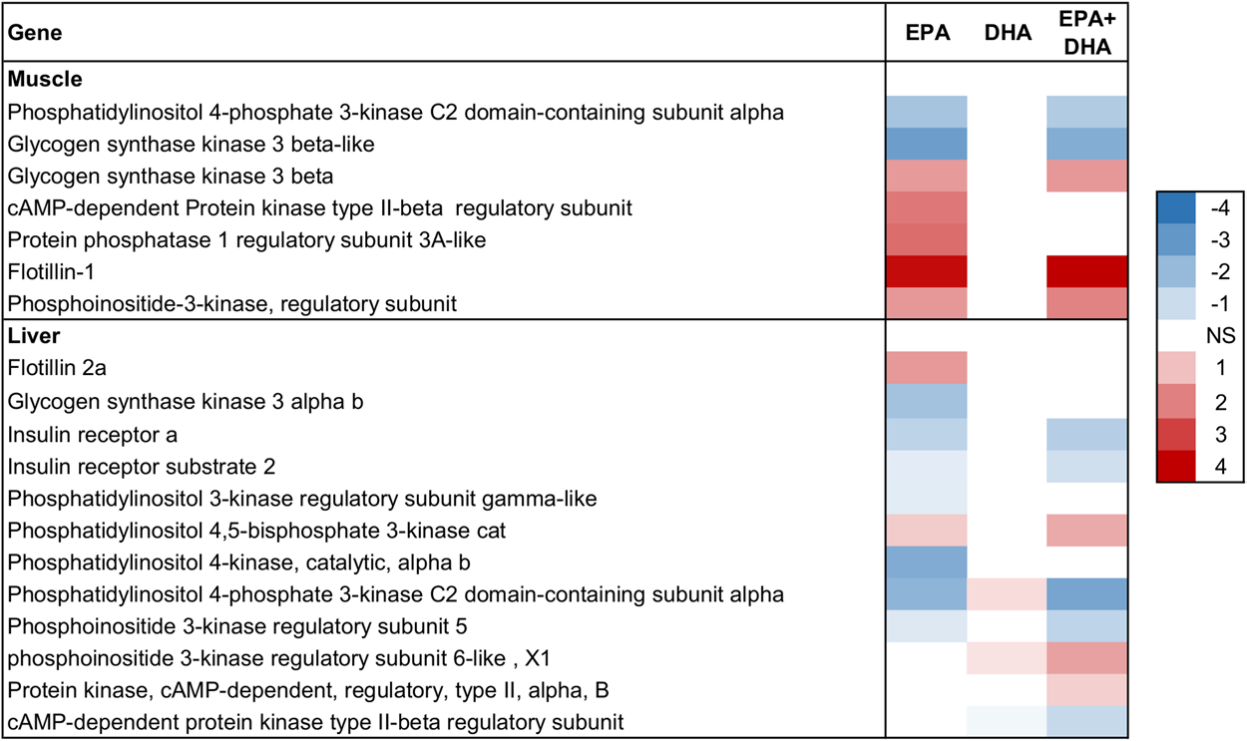
Insulin signaling in muscle and liver. Association between EPA, DHA or EPA + DHA content in muscle and expression of genes in skeletal muscle and liver. Color scale shows linear regression coefficients. NS = no significant association.

**Figure 6:**
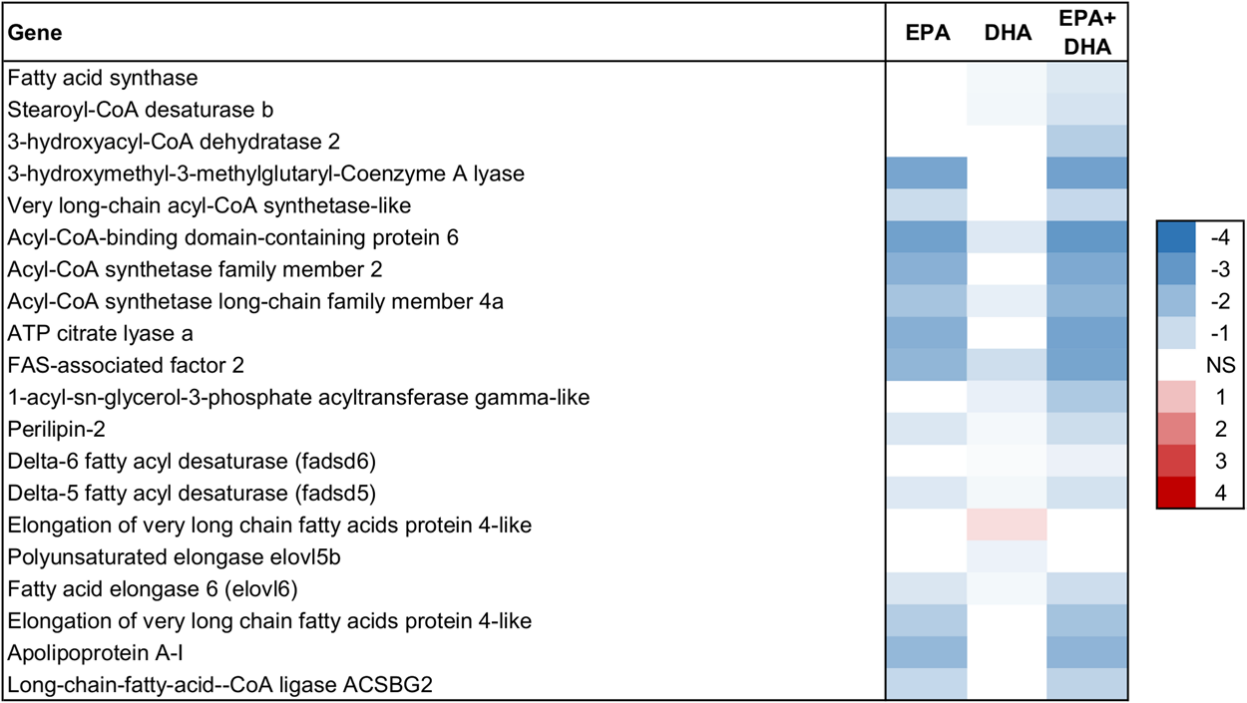
Lipogenesis and fatty acid bioconversion in liver. Association between EPA, DHA or EPA + DHA content in muscle and expression of genes in liver. Color scale shows linear regression coefficients. NS = no significant association.

**Figure 7:**
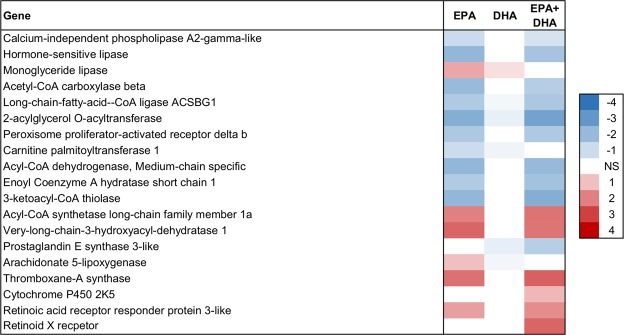
Lipid metabolism in skeletal muscle. Association between EPA, DHA or EPA + DHA content in muscle and expression of genes in skeletal muscle. Color scale shows linear regression coefficients. NS = no significant association.

**Figure 8:**
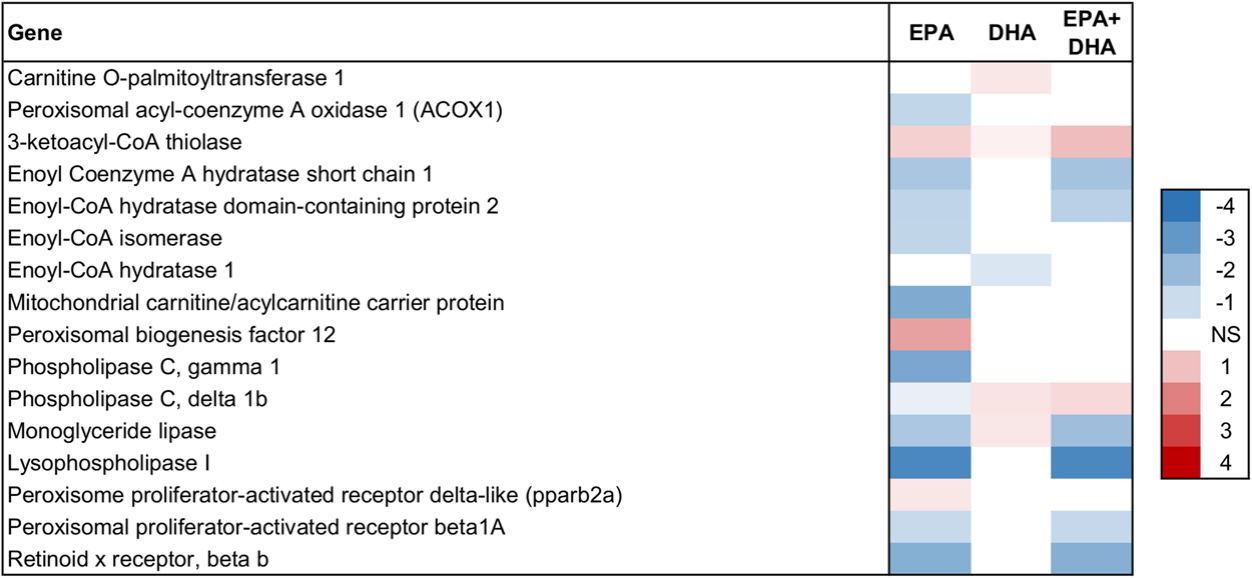
Lipid catabolism in liver. Association between EPA, DHA or EPA + DHA content in muscle and expression of genes in liver. Color scale shows linear regression coefficients. NS = no significant association.

The number of trait-associated genes identified by the linear model association tests was more than 50% higher for the EPA trait than for the DHA trait in both skeletal muscle and liver. In skeletal muscle, the number of trait-associated genes were 3487, 1915 and 3169 for EPA, DHA and EPA + DHA, respectively. The association tests of skeletal muscle FAs with liver gene expression identified 4005 EPA-, 2519 DHA- and 4285 EPA + DHA-associated genes. EPA was not only associated with a higher number of genes, but also had stronger associations compared to DHA (Figs 3–8). This could be related to that EPA is a more bioactive FA compared to DHA, as it is a precursor for many bioactive molecules influencing for instance immunity and inflammatory responses^17^. The transcriptome pattern of skeletal muscle for EPA + DHA was relatively similar to the pattern found for EPA, while DHA was different, most likely because EPA + DHA was more strongly correlated with EPA than DHA (Fig. 1).

Searches for enriched functional classes of GO and KEGG pathways were performed in order to find thematic associations among the trait-associated genes. These identified in total 55 groups enriched for the trait EPA in skeletal muscle and 47 for DHA. In the liver, 62 groups were enriched for skeletal muscle content of EPA and 45 for DHA (Supplementary Table S2). From these, the functional groups considered relevant for the scope of the study were selected (Table 2).

**Table 2:** Enriched functional groups.

Muscle Gene Expression			Liver Gene Expression		
EPA Muscle	Vocabulary	Genes*	EPA Muscle	Vocabulary	Genes*
Citrate cycle (TCA cycle)	KEGG	15/89	Fatty acid beta-oxidation	GO	14/53
Fatty acid metabolism	KEGG	18/109	Lipid metabolic process	GO	44/343
Mitochondrion	GO	266/2005	Mitochondrion	GO	270/2005
Pentose phosphate pathway	KEGG	13/81	Peroxisome	GO	24/140
Insulin signaling pathway	KEGG	54/442	Triglyceride catabolic process	GO	8/34
PPAR signaling pathway	KEGG	21/164	Insulin signaling pathway	KEGG	59/442
Fat cell differentiation	GO	17/129	PPAR signaling pathway	KEGG	36/164
Skeletal muscle tissue development	GO	23/190			
**DHA Muscle**	**Vocabulary**	Genes*	**DHA Muscle**	**Vocabulary**	Genes*
Citrate cycle (TCA cycle)	KEGG	11/89	Biosynthesis of unsaturated fatty acids	KEGG	8/40
Fatty acid metabolism	KEGG	10/109	Fatty acid elongation in mitochondria	KEGG	6/30
Gluconeogenesis	GO	10/90	Lipid metabolic process	GO	37/343
Glycolysis/Gluconeogenesis	KEGG	15/198	Lipid particle	GO	15/88
Mitochondrion	GO	176/2005	Mitochondrion	GO	207/2005
Pyruvate metabolism	KEGG	13/119	PPAR signaling pathway	KEGG	16/164
Skeletal muscle tissue development	GO	17/190			
Striated muscle cell development	GO	6/39			

*Number of genes corresponding to term in the list of trait associated genes/total number of genes of GO/KEGG term.

The enrichment analyses identified several functional groups related to nutrient metabolism as associated with skeletal muscle content of EPA and DHA. The following functional groups were enriched for both FAs; TCA cycle, FA metabolism, mitochondrion and skeletal muscle development (in muscle), lipid metabolic process, peroxisome proliferator activated receptor (PPAR) signalling and mitochondrion (in liver) (Table 2). Functional groups enriched for EPA only included pentose phosphate pathway, insulin and PPAR signalling in muscle, and insulin signalling and lipid catabolic processes in liver. Functional groups enriched for DHA only included glycolysis and gluconeogenesis in muscle, and biosynthesis of unsaturated FAs and FA elongation in liver. No associations were found with protein metabolism. In the following sections, trait-associated genes from these functional groups will be discussed.

It should be noted that the fish in this study was fed a diet rich in EPA and DHA and further fasted for two weeks prior to slaughter, both being factors known to influence nutrient metabolism. Whether the suggested associations of EPA and DHA in Atlantic salmon skeletal muscle with the different metabolic pathways applies to fish fed a commercial diet and with a shorter fasting period prior to slaughter, remains to be elucidated.

### Muscle content of EPA and DHA was associated with skeletal muscle tissue development and function

Several studies on mammals suggest that EPA and DHA can influence the response of skeletal muscle to exercise and nutrient utilization, and improve muscle anabolic processes, although the underlying mechanisms are unclear (reviewed in Jeromson *et al.*^28^).

The enrichment analysis identified skeletal muscle tissue development as an overrepresented group for both EPA and DHA (Table 2). Positive associations with both EPA and DHA were found with expression of the genes *sarcoglycan*, *dystrophin*, *dystroglycan* and *laminin* - all involved in the Dystrophin-Associated Protein Complex (DAPC) in skeletal and cardiac muscle (Fig. 3). The DAPC plays a structural role in the muscle by stabilizing the sarcolemma during contraction and relaxation, and transmits force generated in the muscle sarcomeres to the extracellular matrix^47^.

There was a clear additive effect of EPA and DHA, as shown by the higher regression coefficients of EPA + DHA (Fig. 3). EPA + DHA had an especially strong positive association with expression of *nebulin*. Nebulin is a structural protein that functions in numerous cellular processes including regulation of muscle contraction, improving muscle force efficiency, Z-disc formation, and myofibril assembly^48^.

In conclusion, these results indicate that EPA and DHA are associated with skeletal muscle function and/or development in Atlantic salmon.

### EPA and DHA were associated with carbohydrate metabolism in skeletal muscle

Carnivorous fish have a low capacity to utilize dietary digestible carbohydrates, and have slow blood glucose clearance, however, they do have an active glucose homeostatic system with the presence of almost all the essential biological elements (reviewed in^49^ and^50^).

**Figure 9:**
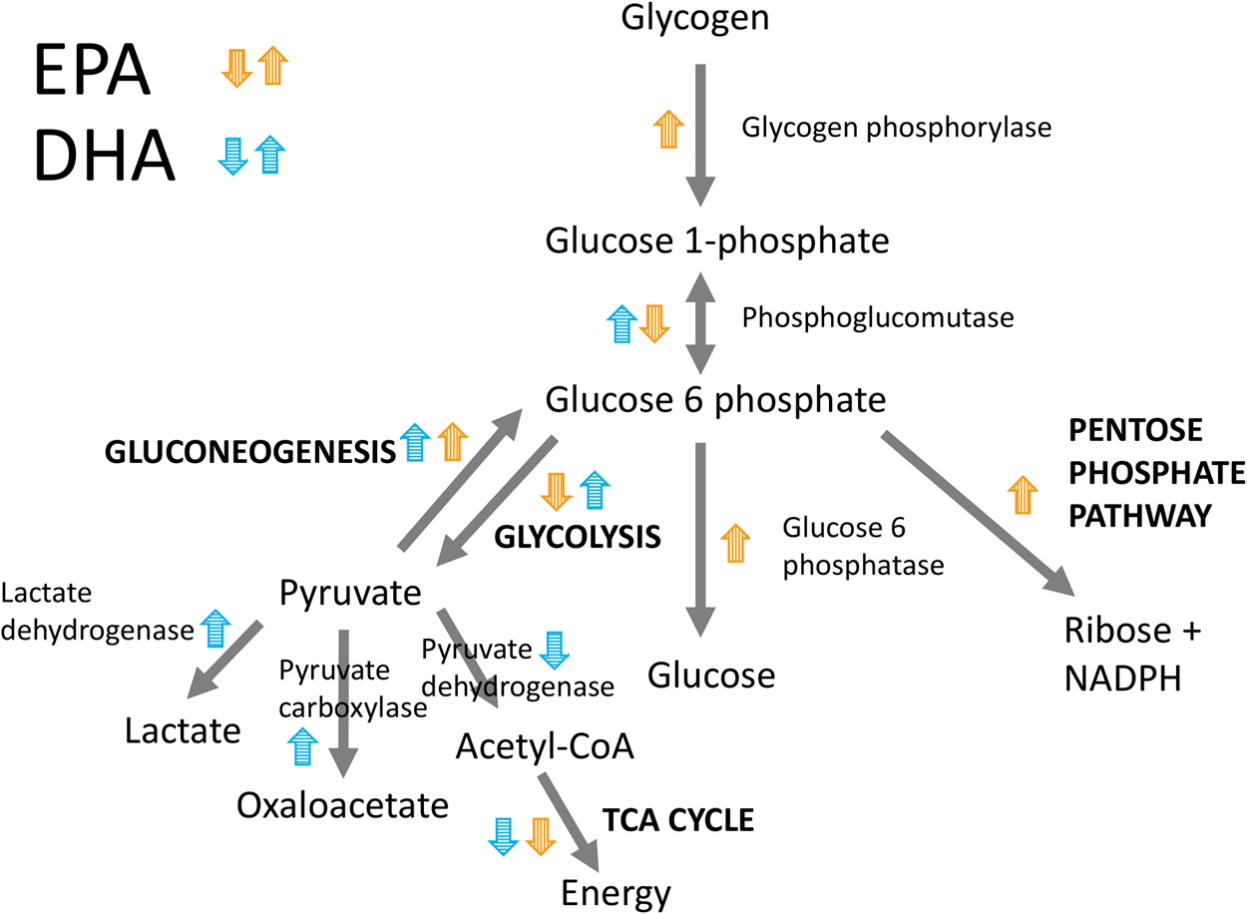
Overview of main results regarding carbohydrate metabolism in skeletal muscle. Colored arrow up and down indicate positive and negative associations, respectively.

EPA and DHA were differently associated with the expression of genes involved in carbohydrate metabolism in the skeletal muscle (Fig. 4). While increasing DHA content was concurrent with increased expression of genes in the glycolytic pathway and the production of pyruvate and lactate (anaerobic glycolysis), EPA was associated with increased expression of genes involved in the pentose phosphate pathway and nucleotide synthesis, in addition to increased glycogen breakdown (Fig. 9).

Carbohydrates are stored as glycogen in the muscle, which can be broken down to glucose units. Gene expression of the catalyzer of the rate-limiting step of glycogen degradation to glucose, *glycogen phosphorylase*, was positively associated with EPA and EPA + DHA (Fig. 4). In addition, EPA was positively associated with expression of the gluconeogenesis-specific gene *glucose-6-phosphatase* that catalyzes the hydrolysis of glucose-6-phosphate to glucose, which can then be released into the blood for use in other tissues. The glucose-6-phosphate derived from the breakdown of glycogen can also be utilized as a substrate for glycolysis, or enter the pentose phosphate pathway to yield ribose derivatives and NADP, which is used in multiple anabolic pathways (Fig. 9).

The results from this study indicate that increased EPA content in skeletal muscle is concurrent with reduced glycolytic activity. Glycolysis and gluconeogenesis are opposing processes, and share several enzymes, acting in reversible reactions. There are three critical stages separating the two pathways. EPA was negatively associated with expression of two of the three glycolysis-specific genes (*hexokinase* and *phosphofructokinase*), and positively associated with expression of the gluconeogenesis-specific gene *glucose-6-phosphatase* that catalyzes the opposite reaction of *hexokinase* (Fig. 4). DHA, on the other hand, was positively associated with gene expression of the glycolysis-specific enzyme *pyruvate kinase* (the last step of glycolysis, forming pyruvate). However, DHA was also positively associated with *pyruvate carboxylase* and *phosphoenolpyruvate carboxykinase*, two gluconeogenesis-specific enzymes that catalyzes the opposite reaction of *pyruvate kinase*. Further, DHA was positively associated with *lactate dehydrogenase*, which converts pyruvate to lactate, and negatively associated with *pyruvate dehydrogenase*, which converts pyruvate to acetyl-coA. Hence, increased DHA content was concurrent with increased anaerobic glycolytic potential, where the pyruvate produced by glycolysis is shuttled towards lactate to a larger degree than to the TCA cycle (Fig. 9).

EPA, however, had a clear negative association with TCA cycle gene expression in muscle, possibly related to the reduced glycolytic activity to form pyruvate; expression of pyruvate dehydrogenase, plus four of the enzymes catalyzing TCA cycle, were negatively associated with EPA. The only TCA-cycle gene whose expression was positively associated with EPA content was *α-ketoglutarate dehydrogenase* (Fig. 4).

The enrichment analysis suggested an association between the pentose phosphate pathway (PPP) and EPA (Table 2). The regression coefficients showed that *ribose-phosphate pyrophosphokinase 1* (*PRPS1*) expression was positively associated with EPA content in the muscle (Fig. 4). *PRPS1* catalyzes the synthesis of phosphoribosylpyrophosphate (PRPP) that is essential for nucleotide synthesis. This may indicate that EPA stimulates nucleotide synthesis in muscle, although the cause-effect relationship is unknown. Nucleotides participate in nearly all biochemical processes in the body as for instance precursors for DNA and RNA, or energy, metabolic regulators, coenzymes and activated intermediates in biosynthesis^51^. The results therefore suggest that in a skeletal muscle rich in EPA, rather than entering glycolysis, the glucose-6-phosphate from breakdown of glycogen seems to be released as glucose, or shuttled into the PPP.

In conclusion, this study indicates that muscle EPA and DHA content has apparent links with carbohydrate metabolism in Atlantic salmon, as has previously been demonstrated in mammals.

### Insulin regulation in Atlantic salmon and its association with EPA and DHA

Skeletal muscle is a major site of insulin action, and muscle LC n-3 PUFA effects on insulin sensitivity has been observed in mice^52^, pigs^53,54^ and human^23,24^. Carnivorous fish possess an insulin-signaling system similar to mammals, although its role is not completely clarified^49,50^. In Atlantic salmon adipose tissue, insulin improves the differentiation capacity of preadipocytes towards mature adipocytes, similar to what happens in human adipose tissue^55^.

The enrichment analysis identified the insulin-signaling pathway as an enriched group for EPA, but not DHA, in both skeletal muscle and liver (Table 2), and expression of several genes related to insulin were associated with EPA content in skeletal muscle. In skeletal muscle, expression of the two genes *phosphatidylinositol 4-phosphate 3-kinase C2 domain-containing subunit alpha-like*, and *phosphatidylinositol 3-kinase regulatory subunit beta-like*, in the phosphoinositide 3-kinase (PI3K) family were positively associated with EPA content (Fig. 5). PI3-kinases are known to play key roles in many signaling pathways in mammals^56,57^, including glucose uptake and insulin signaling^58,59^. Further, expression of *cAMP-dependent protein kinase type II-beta regulatory subunit-like*, which belongs to the protein kinase A (PKA) family of enzymes, was also positively associated with EPA. Studies in mammals have shown that a correct regulation of cAMP/PKA activity is crucial for glucose homeostasis and skeletal muscle insulin sensitivity^60^. The gene expression of *flotillins*, which are considered as markers of lipid rafts and implicated in numerous signaling events, including insulin signaling^61^, increased in both skeletal muscle and liver with increasing content of EPA in muscle (Fig. 5).

These results indicate that EPA is involved in insulin regulation in Atlantic salmon. This is in agreement with what was previously demonstrated in mammals^25,52,62^.

### High skeletal muscle content of EPA and DHA was negatively associated with hepatic lipogenesis

Lipogenesis is the formation of lipids, and includes formation of triglycerides for storage as well as *de novo* lipogenesis of FAs. The crucial carbon source for *de novo* lipogenesis is acetyl-CoA formed in mitochondria from the oxidative decarboxylation of pyruvate or the oxidative degradation of some amino acids.

The analysis of the liver transcriptome showed that both EPA and DHA were associated with reduced expression of genes involved in lipogenesis (Fig. 6). For example, several *acyl-CoA synthethases*, *fatty acid synthase (FAS)*, *FAS-associated factor 2*, as well as *1-acylglycerol-3-phosphate acyltransferase gamma-like* (*GPAT1)*, a key enzyme for hepatic triglyceride synthesis. Several studies have shown reduced hepatic triglyceride synthesis with increasing dietary levels of EPA and/or DHA, both in mammals^63,64^ and salmonid fish including rainbow trout^65^ and Atlantic salmon^66^. Our study found an association between reduced lipogenesis and increased EPA in the skeletal muscle tissue of fish that received the same feed.

Although the cause-effect relationship cannot be determined, these results may indicate that increasing the skeletal muscle EPA and DHA content lowers the risk of developing fatty liver, which may promote health as fatty liver is recognized as a health problem in farmed Atlantic salmon^67^. However, this effect was not seen on the phenotype of the fish; the correlation between liver fat percentage and skeletal muscle EPA and DHA content was close to zero (Supplementary Fig. S1).

In skeletal muscle, expression of the key genes involved in lipogenesis were negatively associated with EPA, for instance *2-acylglycerol O-acyltransferase 1* that catalyzes the formation of diacylglycerol, and *acetyl CoA carboxylase* (ACC) that is involved in regulation of fatty acid synthesis (Fig. 7). These results indicate a possible inhibitory effect of EPA on lipogenesis in Atlantic salmon skeletal muscle, which has not been reported before.

### Lipid catabolism decreased with increasing EPA in skeletal muscle

Fatty acid catabolism is the major source of energy in salmonid fish. The process is termed β-oxidation, and occurs in the mitochondria and peroxisomes^68^. In Atlantic salmon, β-oxidation is an important source of energy in several tissues^5,69,70^. Increased EPA content in skeletal muscle was negatively associated with expression of lipid catabolic genes in both liver and skeletal muscle (Figs 7 and 8). DHA had weak associations compared to EPA.

In the liver, we observed that EPA was associated with reduced expression of the lipid catabolic genes *monoglyceride lipase* and *phospholipase C* (Fig. 8), as well as e*noyl-CoA hydratase* and *Delta(3*,*5)-Delta(2*,*4)-dienoyl-CoA isomerase*, which are both involved in β-oxidation. Expression of the mitochondrial β-oxidation biomarker *carnitine palmitoyltransferase 1 (CPT1)*, was also negatively associated with EPA.

In skeletal muscle, EPA and EPA + DHA were negatively associated with expression of three genes coding for β-oxidation enzymes: *3-ketoacyl-CoA thiolase*, *medium-chain specific acyl-CoA dehydrogenase* and *enoyl Coenzyme A hydratase* (Fig. 7). In addition, expression of *PPAR β/δ*, a central regulator of energy metabolism that stimulates genes of β-oxidation^71,72^, and *CPT1*, had negative associations with EPA.

Mitochondrial β-oxidation capacity is determined by many factors, for instance mitochondrial biogenesis and function in general. Expression of a number of mitochondrial genes were associated with EPA and DHA content in skeletal muscle, both positively and negatively, thus, the overall effect was difficult to determine, although the majority of the associations were negative (Table 3).

**Table 3:** Genes classified in the GO functional group *Mitochondrion* that were significantly associated with the fatty acid traits.

Trait	Total no. of associated genes	Negatively associated	Positively associated	Mean regression coefficient
**Muscle**				
EPA	268	154	114	−0.06
DHA	176	128	48	−0.19
EPA + DHA	192	116	76	−0.11
**Liver**				
EPA	265	215	50	−1
DHA	206	165	41	−0.28
EPA + DHA	259	208	51	−1.21

Increased EPA in the diet generally increases the content of EPA in both liver and skeletal muscle, and has been reported to result in increased FA oxidation in liver tissue in mammals^73–76^. In Atlantic salmon, however, there are varying results from feeding trials regarding the effect of high EPA and DHA on hepatic β-oxidation capacity^77–79^. Regarding skeletal muscle, Aas *et al*.^23^ reported that preincubation of human skeletal muscle cells with EPA decreased FA oxidation, which concurred with increased incorporation of EPA into complex cellular lipids. Thus, the variation in β-oxidation capacity observed in this study may reflect on the ratio between utilization of EPA for energy production and its deposition in skeletal muscle.

Overall, expression of genes involved in lipid catabolism decreased in both liver and skeletal muscle with increasing content of EPA and EPA + DHA in skeletal muscle.

### Eicosanoids, resolvins and protectins

In the muscle, genes related to conversion of long chain FAS were positively associated with EPA and EPA + DHA. These included *very-long-chain-3-hydroxyacyl-dehydratase 1*, which catalyzes the third of the four reactions of the long-chain fatty acids elongation cycle, as well as *long-chain Acyl-CoA synthetase* (Fig. 7). This indicates further bio-conversion of EPA and DHA to very long chain n-3 PUFAs and possibly formation of resolvins and protectins. It has been suggested that resolvins and protectins are mediators of the beneficial effects of EPA on insulin signaling^80,81^. Expression of a few eicosanoid-related genes, such as *Arachidonate 5-lipoxygenase (ALOX)* and *Thromboxane-A synthase*, were positively associated with EPA (Fig. 7). Further, *Cytochrome P450* was positively associated with EPA + DHA. ALOX metabolizes EPA to 5-hydroperoxy-EPA which is then converted to 5-series of Leukotriene products^82^. ALOX is also known to cooperate with other lipoxygenase and cyclooxygenase cytochrome P450 enzymes in metabolic pathways that metabolize EPA to resolvins of the E series^82^. These results indicate that EPA and DHA contents of muscle are associated with skeletal muscle production of eicosanoids and resolvins.

### What determines EPA and DHA content in skeletal muscle?

Individual differences in EPA and DHA content were observed in fish of similar size fed an identical diet. This individual variation may result from many factors, for instance individual differences in FA deposition, β-oxidation of FAs, omega-3 bioconversion in skeletal muscle, or uptake of these FAs from blood, which again can be influenced by omega-3 bioconversion in the liver and intestine, which are the major sites of omega-3 bioconversion in Atlantic salmon^5^.

In the current study, the hepatic expression of the fatty acid desaturase genes *fadsd5* and *fadsd6* was negatively associated with skeletal muscle level of EPA and DHA (Fig. 6). In the skeletal muscle, no desaturases or elongases involved in the omega-3 bioconversion pathway showed any association with either EPA or DHA (although *fadsd5*, *fadsd6* and *elovl5* were expressed in skeletal muscle tissue). This may point to factors other than omega-3 bioconversion which are more important for determining the EPA and DHA content in skeletal muscle. Albeit we cannot determine cause and effect based on the association analyses, our results point to reduced β-oxidation as a possible determining factor for EPA and the sum of EPA + DHA, since the individuals with the highest EPA and EPA + DHA content had a lower expression of β-oxidation genes. Studies in Atlantic salmon have shown that LC n-3 PUFAs given in surplus are oxidized for the production of energy^83^. Thus, our results suggest that the reason why some individuals have a higher level of EPA and DHA in the skeletal muscle is that these FAs are deposited rather than oxidized for energy.

## Conclusions

The current study shows that there are big individual variations in EPA and DHA content of skeletal muscle between Atlantic salmon fed the same diet, and that these variations are associated with differences in expression of genes involved in nutrient metabolism. The main results are:

- Correlations between liver and skeletal muscle were very low regarding both EPA and DHA content.
- The correlation between EPA and DHA percentage in skeletal muscle was low, and over 50% more genes were associated with EPA than DHA. Also, the two FAs were associated with different gene expression profiles. This information is relevant for consideration of future feed production strategies, since several of new alternative feed ingredients contain either EPA or DHA, not both.
- Skeletal muscle content of EPA and DHA was positively associated with muscle tissue development and function.
- Skeletal muscle content of EPA and DHA was associated with carbohydrate metabolism. While increasing DHA content was concurrent with increased expression of genes in the glycolytic pathway and the production of pyruvate and lactate, EPA was associated with increased activity of the pentose phosphate pathway and nucleotide synthesis in addition to increased glycogen breakdown. Furthermore, skeletal muscle content of EPA, but not DHA, was associated with expression of genes involved in insulin signaling in both skeletal muscle and liver tissues.
- EPA and DHA were associated with expression of genes involved in eicosanoid and resolvin production.
- EPA was negatively associated with expression of genes involved in lipid catabolism, in both liver and muscle. Thus, a possible reason why some individuals have a higher level of EPA in the skeletal muscle is that they deposit - rather than oxidize - EPA for energy.

## Supplementary information

Figure S1

S1 Table.

S2 Table.

## Data Availability

The datasets generated and analyzed during the current study are not publicly available since it contains commercially sensitive data, but are available from the corresponding author on reasonable request.
