# Supplementary material for: Individual differences in EPA and DHA content of Atlantic salmon are associated with gene expression of key metabolic processes
Source: Sci Rep. 2019 Mar 7;9:3889. doi: 10.1038/s41598-019-40391-2 (PMC6405848; doi:10.1038/s41598-019-40391-2)

Individual differences in EPA and DHA content of Atlantic salmon are associated with gene expression of key metabolic processes

Siri S. Horn^1,2^*, Anna K. Sonesson^1^, Aleksei Krasnov^1^, Hooman Moghadam^3^, Borghild Hillestad^3^, Theo H.E. Meuwissen^2^, Bente Ruyter^1^

^1^ Nofima (Norwegian institute of Food, Fisheries and Aquaculture research), PO Box 210, N-1432 Ås, Norway

^2^ Department of Animal and Aquaculture Sciences, Norwegian University of Life Sciences, N-1430 Ås, Norway

^3^ SalmoBreed AS, Sandviksboder 3A, N-5035 Bergen, Norway

* Corresponding author

E-mail: siri.storteig.horn@nofima.no

**Supplementary Figure 1.** Correlation between liver fat content and contents of (A) EPA, (B) DHA and (C) EPA+DHA in skeletal muscle.


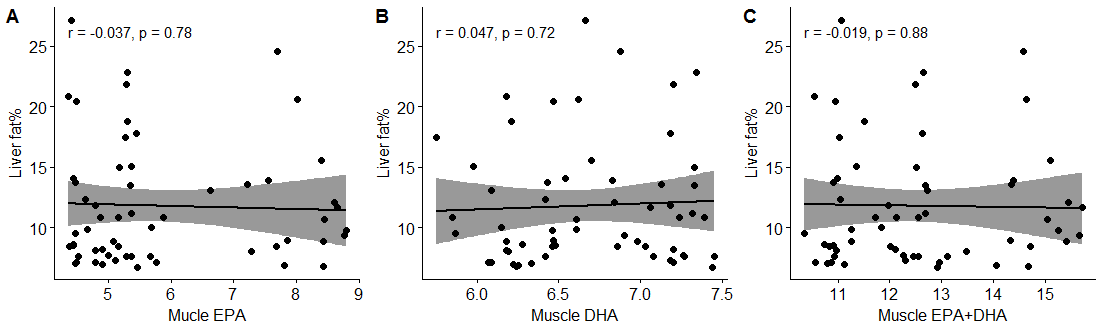

Supplement: Supplementary file 1 — Figure S1 [file 41598_2019_40391_MOESM1_ESM.docx]
